# Association Between Recombinant Growth Hormone Therapy and All-Cause Mortality and Cancer Risk in Childhood: Systematic Review and Meta-Analysis

**DOI:** 10.3389/fped.2022.866295

**Published:** 2022-04-22

**Authors:** Mengyang He, Xiangling Deng, Xuan Wang, Yuxiang Wan, Jinchang Huang, Zhixin Zhang, Wenquan Niu

**Affiliations:** ^1^Graduate School, Beijing University of Chinese Medicine, Beijing, China; ^2^Beijing University of Chinese Medicine Third Affiliated Hospital, Beijing, China; ^3^Department of International Medical, China-Japan Friendship Hospital, Beijing, China; ^4^Institute of Clinical Medical Sciences, China-Japan Friendship Hospital, Beijing, China

**Keywords:** mortality, children, cancer, medication safety, rhGH

## Abstract

**Objectives:**

The safety of recombinant human growth hormone (rhGH) treatment in childhood and the role of rhGH therapy in promoting tumorigenesis and progression have been the subject of debate for decades. We aimed to systematically assess the relationship between rhGH therapy in children and adolescents and clinical outcomes, including all-cause mortality, cancer mortality, cancer incidence, and risk of the second neoplasm.

**Methods:**

Literature retrieval, study selection, and data extraction were completed independently and in duplicate. Effect-size estimates are expressed as standardized mortality ratios (SMRs), standardized incidence ratio (SIR), and relative risk (RR) with a 95% CI.

**Results:**

Data from 24 articles, involving 254,776 persons, were meta-analyzed. Overall analyses revealed the association of rhGH therapy was not statistically significant with all-cause mortality (SMR = 1.28; 95% CI: 0.58–2.84; *P* = 0.547; *I*^2^ = 99.2%; Tau^2^ = 2.154) and cancer mortality (SMR = 2.59; 95% CI: 0.55–12.09; *P* = 0.228; *I*^2^ = 96.7%; Tau^2^ = 2.361) and also cancer incidence (SIR = 1.54; 95% CI: 0.68–3.47; *P* = 0.229; *I*^2^ = 97.5%; Tau^2^ = 2.287), yet statistical significance was observed for second neoplasm (RR = 1.77; 95% CI: 1.33–2.35; *P* = 0.001; *I*^2^ = 26.7%; Tau^2^ = 0.055). Differences in the geographic region, gender, treatment duration, mean rhGH dose, overall rhGH exposure dose, and initial disease accounted for heterogeneity in the subgroup analyses.

**Conclusion:**

Our findings indicate that the rhGH therapy is not related to all-cause mortality and cancer mortality and cancer incidence, yet it seems to trigger a second tumor risk. Future prospective studies are needed to confirm our findings and answer the more challenging question regarding the optimal dose of rhGH therapy in children and adolescents.

## Introduction

Since 1957, human growth hormone has been used to treat growth hormone deficiency and short stature, and it was supplanted by recombinant human growth hormone (rhGH) in 1985 (1). Initially, growth hormone was prescribed to patients with a severe growth hormone deficiency and its application is currently extended to children with short stature that is not primarily caused by an endogenous growth hormone deficiency, as well as to other scenarios, such as small for gestational age without catch-up growth or idiopathic short stature, Turner syndrome, short stature homeobox gene deficiency, Noonan syndrome, Prader–Willi syndrome, and growth failure associated with chronic renal insufficiency (2, 3). Generally, growth hormone therapy is considered to be safe, and serious adverse reactions rarely occur (4–6). However, in recent decades, the potential link between growth hormone therapy and tumor development or recurrence has gained increasing attention in clinical practice (7–11). In 2014, Deodati et al. (12) have undertaken a meta-analysis and reported that patients treated with growth hormone during childhood and adolescence had a significantly increased risk of all-cause mortality, cancer incidence, and second neoplasm after primary cancer. Contrastingly, in the to-date largest long-term follow-up study by Sävendahl et al., rhGH therapy was not associated with all-cause mortality in patients with isolated growth hormone deficiency or idiopathic short stature (13), and another large cohort study by Child et al. (2) also reported no significant association. In this context, the association between growth hormone therapy and all-cause mortality is still subject to an ongoing debate. With the accumulating publications afterward, there is a need to reexamine this association in a more comprehensive manner.

In an attempt to address this need and derive more reliable estimates, we performed an updated meta-analysis by pooling the results of both the prospective and retrospective cohorts in the medical literature to examine the association of rhGH therapy in children and adolescents with multiple clinical outcomes, including all-cause mortality, cancer mortality, cancer incidence, and risk of the second neoplasm. Another attempt was to identify the reasons for previous inconsistent reports, in other words, between-study heterogeneity.

## Methods

The performance of the meta-analysis has adhered to the guidelines in the Preferred Reporting Items for Systematic Reviews and Meta-analyses (PRISMA) statement (14). The PRISMA checklist is given in Supplementary Table 1.

This study is a meta-analysis of published studies; hence, ethical approval and informed consent are not needed.

### Search Strategy

A literature search was conducted by reviewing the PubMed, MEDLINE, EMBASE, and Web of Science databases as of 6 November 2021. The following medical topic terms were used: (growth hormone or human growth hormone or somatotropin or somatropin or somatotrophin or GH or hGH or rhGH or rhGH or GH deficiency or growth hormone replacement therapy or GH replacement therapy) [Title] and (mortality or death or fatal or fatality or cancer or cancers or neoplasia or neoplasias or neoplasm or tumors or tumor or malignancy or malignancies or malignant neoplasm or CVD or cardiovascular disease) [Title/Abstract]. The reference lists of major retrieved articles were also manually searched to avoid potential missing hits.

The search process was independently conducted by two investigators (MH and XD) using the same medical topic terms. All the references retrieved were combined and duplicates were removed.

### Inclusion/Exclusion Criteria

Our analysis was restricted to articles that met the following criteria: (1) study participants: women with BC; (2) endpoints: standardized mortality ratios (SMRs) or standardized incidence ratio (SIR) or relative risk (RR) with 95% CI; (3) study type: retrospective or prospective cohorts; (4) baseline exposure: growth hormone therapy; (5) follow-up rate: at least 70%; and (6) follow-up duration: ≥1 year. Articles were excluded if the involved study participants were adults or if they are case reports or case series, editorials, and narrative reviews.

### Data Extraction

Two investigators (MH and XD) independently extracted data from each qualified article, including the first author, year of publication, the country where the study was conducted, sample size, study design, age at start rhGH therapy, rhGH dose, treatment duration, initial diagnosis, treatment duration, mean rhGH dose, overall exposure, effect estimation, and other confounding risk factors, if available. The divergence was resolved through a joint reevaluation of original articles, and if necessary, by a third author (WN).

### Statistical Analyses

Data management was handled using the STATA software version 14.1 for Windows (Stata Corporation, College Station, Texas, USA). Effect-size estimates are expressed as SMR, SIR, or RR with 95% CI, where appropriate, and they are derived under the Mantel–Haenszel model. The difference between the two estimates was tested by the *Z*-test, as proposed by Altman and Bland (15). Pooled effect-size estimates were derived under a random-effects model, irrespective of the magnitude of between-study heterogeneity.

The inconsistency index (*I*^2^) statistic, which represents the percent of diversity that is due to heterogeneity rather than chance, is used to quantify the magnitude of heterogeneity that was derived from a random-effects Mantel–Haenszel model. The *I*^2^ >50% indicates the presence of significant heterogeneity and a higher percent corresponds to a higher degree of heterogeneity. Besides *I*^2^ statistic, another index, τ^2^ (Tau^2^), was used to explore the sensitivity of the results to different levels of between-study heterogeneity. To account for possible sources of between-study heterogeneity from clinical and methodological aspects, a panel of prespecified subgroup analyses were performed according to geographic region, published year, study design, age at start rhGH therapy, rhGH dose, treatment duration, initial diagnosis, mean rhGH dose, exposure, and follow-up interval, respectively.

The likelihood of publication bias was evaluated by both Begg's funnel plots and Egger's regression asymmetry tests at a significance level of 10%. The trim-and-fill method was also used to speculate the number of theoretically missing studies.

## Results

### Eligible Studies

**Figure 1:**
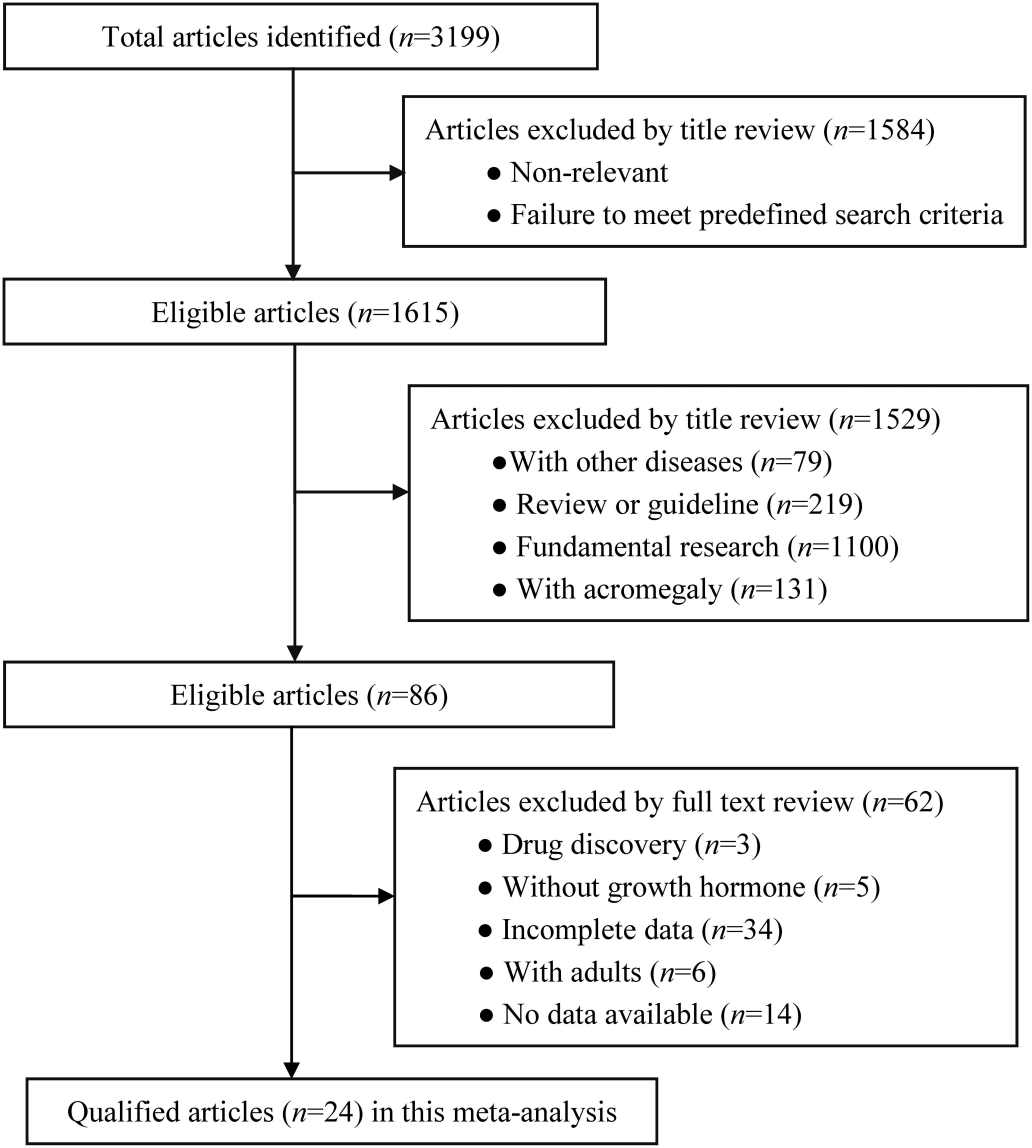
Flowchart of records retrieved, screened, and included in this meta-analysis.

A total of 3,199 articles were initially identified after searching predefined public datasets according to subject terms, of which 24 met our eligibility criteria, including 2,54,776 children and adolescents. The detailed selection process is shown in Figure 1. Among the eligible articles included, effect size estimates are expressed as SMR, SIR, and RR with 95% CIs.

### Study Characteristics

Supplementary Table 2 shows the baseline characteristics of all the qualified articles in this meta-analysis. Of the 24 articles included, the outcome measure was all cause SMR in 7 articles (2, 13, 16–20), cancer SIR in 12 articles (1, 2, 5, 17, 19, 21–27), cancer SMR in 4 articles (16, 21, 25, 27), and second neoplasm in 8 articles (9, 10, 28–33). Only two articles (13, 16) presented data separately in boys and girls. Overall exposure of rhGH therapy was classified into four categories: <25, 25–50, 50–100, and ≥100 mg/kg.

Based on the previous medical history and physical health status, 3 articles (13, 16, 19) divided children into the low-risk, moderate-risk, and high-risk groups and 4 articles (5, 21, 22, 25) assorted children into the not-high-risk group.

Of the 24 qualified articles, two (13, 16) articles evaluated rhGH therapy duration <5 and ≥5 years. In total, eighteen articles were retrospective in design (5, 9, 10, 16, 18, 19, 21–24, 26–33) and 6 articles were prospective (1, 2, 13, 17, 20, 25). All the eligible articles were classified geographically into North America (5, 9, 10, 28), Asia (19), Europe (1, 13, 16–18, 21, 27, 29, 32, 33), and multinational (2, 20, 22–26, 30, 31).

### Quality Assessment

Supplementary Table 3 shows the quality assessment of all the qualified articles by using the Newcastle–Ottawa Scale (NOS) tool for cohort studies. The average total score was 7.46 (range: 7–8), with an SD of 0.5.

### Overall Analyses

**Figure 2:**
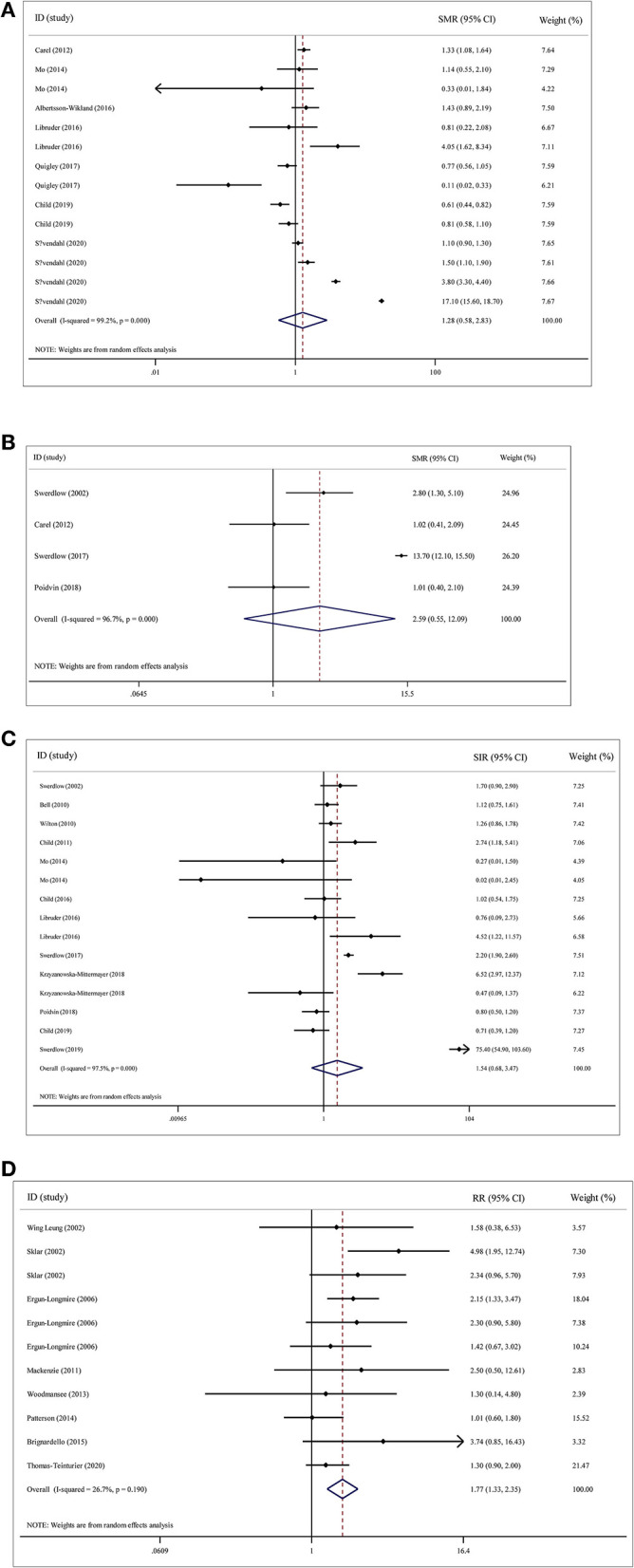
Overall analyses on the association of recombinant human growth hormone (rhGH) therapy with mortality and cancer risk. **(A)** rhGH therapy and all-cause mortality. **(B)** rhGH therapy and cancer mortality. **(C)** rhGH therapy and cancer incidence. **(D)** rhGH therapy and second neoplasm.

After pooling the results of all the qualified articles, there was no statistical significance between rhGH therapy in childhood and all-cause mortality (SMR = 1.28; 95% CI: 0.58–2.84; *P* = 0.547; *I*^2^ = 99.2%; *Tau*^2^ = 2.154), cancer mortality (SMR = 2.59; 95% CI: 0.55–12.09; *P* = 0.228; *I*^2^ = 96.7%; *Tau*^2^ = 2.361), and standardized incidence ratio for cancer (SIR = 1.54; 95% CI: 0.68–3.47; *P* = 0.229; *I*^2^ = 97.5%; *Tau*^2^ = 2.287). In contrast, there was a statistically significant association with the development of second neoplasm (RR = 1.77; 95% CI: 1.33–2.35; *P* = 0.001; *I*^2^ = 26.7%; *Tau*^2^ = 0.055) (Figure 2).

### Cumulative and Influential Analyses

In the cumulative analyses, included studies got completely similar conclusions consistently and trends tended to stabilize. The influential analyses revealed no significant impact of any single study on overall effect-size estimates.

### Publication Bias

**Figure 3:**
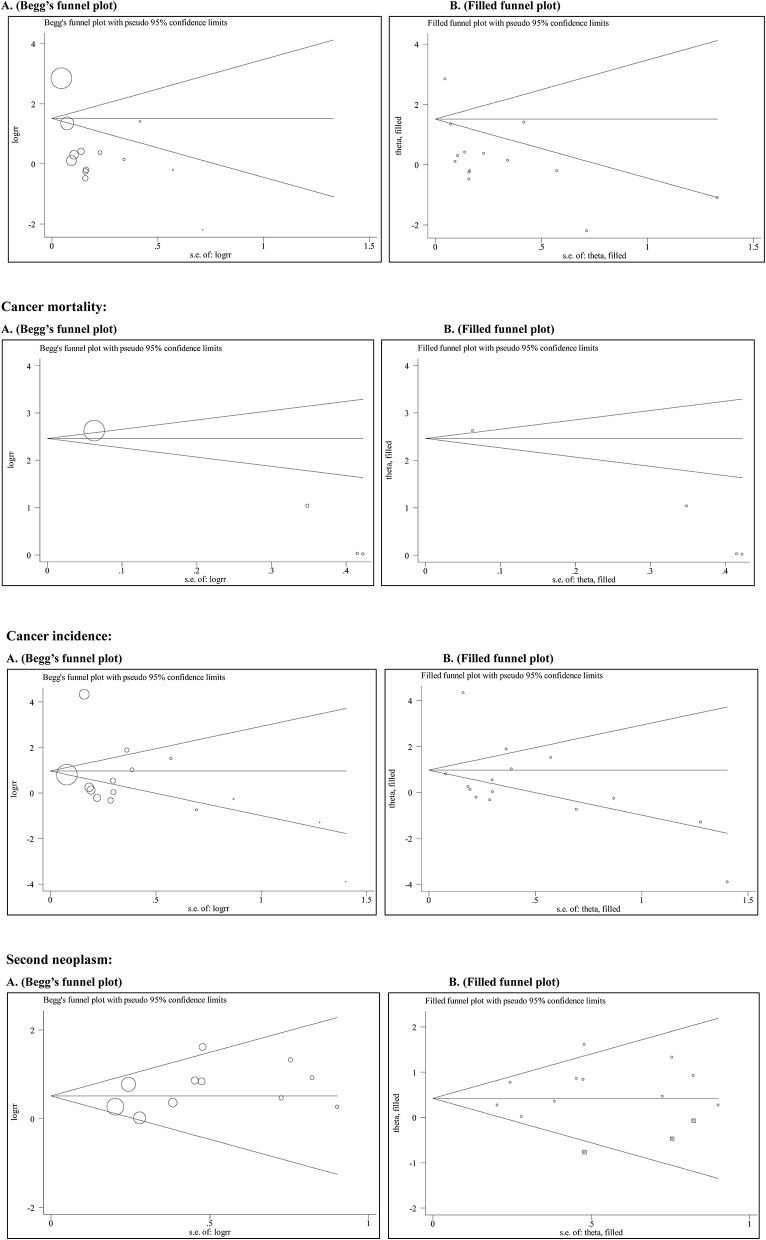
The Begg's and filled funnel plots for the association of rhGH therapy with mortality and cancer risk. All-cause mortality: **(A)** Begg's funnel plot, **(B)** Filled funnel plot. Cancer mortality: **(A)** Begg's funnel plot, **(B)** Filled funnel plot. Cancer incidence: **(A)** Begg's funnel plot, **(B)** Filled funnel plot. Second neoplasm: **(A)** Begg's funnel plot, **(B)** Filled funnel plot.

Figure 3 shows Begg's funnel plot and Egger's test for assessing publication bias of rhGH therapy with all-cause mortality, cancer mortality, the standardized incidence of cancer, and the occurrence of the second neoplasm.

Begg's funnel plots seemed symmetrical. As reflected by Egger's test, there was a low likelihood of publication bias for standardized incidence of cancer (*P* = 0.525) and occurrence of second neoplasm (*P* = 0.167). Further investigations using the “trim and fill” method showed that 3 theoretically missing studies were required to make Begg's funnel plot symmetrical for the occurrence of the second neoplasm. However, no study was required in theory for standardized incidence of cancer.

There was statistical evidence of asymmetry by using Eggers's tests in all-cause mortality (*P* = 0.015) and cancer mortality (*P* = 0.008). The “trim and fill” method did not produce any derivations from the original estimates.

### Subgroup Analyses

A series of prespecified subgroup analyses were conducted to account for possible sources of between-study heterogeneity for rhGH therapy with the all-cause mortality, cancer mortality, the standardized incidence of cancer, and the occurrence of second neoplasm (Table 1).

**Table 1:** Overall and subgroup analyses on the association of recombinant human growth hormone therapy with mortality and cancer risk.

**Group**	**Number of qualified studies**	**Mortality or cancer risk**		***Tau* ^2^**
		**RR (95% CI); *P***	***I* ^2^**	
**Overall analyses**				
All cause SMR	14	1.28 (0.58–2.84); 0.547	99.2%	2.154
Cancer SMR	4	2.59 (0.55–12.09); 0.228	96.7%	2.361
Cancer SIR	15	1.54 (0.68–3.47); 0.229	97.5%	2.287
RR SN	11	1.77 (1.33–2.35); 0.001	26.7%	0.055
**Subgroup analyses based on mortality or cancer risk**				
**By region based on All cause SMR**				
Europe	8	1.92 (0.71–5.23); 0.202	99.4%	1.938
Asia	2	1.90 (0.39–9.17); 0.424	80.6%	1.044
International	4	0.66 (0.47–0.92); 0.016	65.1%	0.070
**By region based on Cancer SMR**				
Europe	3	1.47 (0.73–2.96); 0.279	59.3%	0.227
**By region based on Cancer SIR**				
Europe	5	1.09 (0.08–14.50); 0.951	98.8%	8.071
Asia	7	2.09 (0.37–11.81); 0.058	65.8%	1.046
International	2	1.59 (0.98–2.57); 0.404	85.3%	0.314
**By region based on RR SN**				
North America	6	2.20 (1.61–3.02); <0.001	0.0%	0.000
Europe	3	1.57 (0.93–2.66); 0.094	12.9%	0.051
International	2	1.03 (0.61–1.75); 0.904	0.0%	0.000
**By gender based on All cause SMR**				
Boys	5	2.50 (0.81–7.69); 0.110	99.4%	1.629
Girls	5	3.01 (0.71–12.78); 0.135	99.2%	2.663
**By study design based on All cause SMR**				
Prospective	10	1.16 (0.44–3.10); 0.765	99.4%	2.334
Retrospective	4	1.56 (1.02–2.38); 0.041	60.6%	0.102
**By study design based on Cancer SMR**				
Retrospective	3	1.47 (0.73–2.96); 0.279	59.3%	0.227
**By study design based on Cancer SIR**				
Prospective	5	1.22 (0.15–10.31); 0.853	99.1%	5.286
Retrospective	10	1.53 (1.02–2.31); 0.040	75.8%	0.286
**By study design based on RR SN**				
Retrospective	11	1.77 (1.33–2.35); <0.001	26.7%	0.055
**By risk based on All cause SMR**				
Low	54	1.25 (1.17–1.34); <0.001	14.4%	0.009
Moderate	22	4.00 (3.50–4.57); <0.001	69.0%	0.061
High	21	16.88 (14.52–19.63); <0.001	90.1%	0.101
**By risk based on Cancer SMR**				
Not high	4	8.28 (1.62–42.41); 0.011	99.6%	2.714
**By risk based on Cancer SIR**				
Not high	6	1.88 (0.99–3.57); 0.055	96.9%	0.602
**By GH treatment duration (years) based on All–cause SMR**				
<5	19	3.20 (1.78–5.76); <0.001	98.2%	1.665
≥5	8	1.96 (0.83–4.65); 0.126	95.8%	1.427
**By overall GH exposure dose (mg/kg) based on All cause–SMR**				
<25	7	2.03 (0.62–6.59); 0.241	98.8%	2.493
25–50	5	2.85 (0.89–9.09); 0.077	98.3%	1.711
50–100	4	2.64 (0.81–8.55); 0.106	96.9%	1.341
≥100	4	3.32 (1.22–9.08); 0.019	85.8%	0.832
**By follow up (years) based on All cause-SMR**				
≥10	11	0.98 (0.75–1.29); 0.899	78.3%	0.127
<10	3	4.62 (1.19–18.01); 0.027	99.6%	1.435
**By follow up (years) based on Cancer SMR**				
≥10	4	2.59 (0.55–12.09); 0.228	96.7%	2.361
**By follow up (years) based on Cancer SIR**				
≥10	15	1.54 (0.68–3.47); 0.299	97.5%	2.287
**By follow up (years) based on RR SN**				
≥10	11	1.77 (1.33–2.35); <0.001	26.7%	0.055

*RR, risk ratio; 95% CI, 95% confidence interval, SMR, standardized mortality ratios; SIR, standardized incidence ratio; SN, second neoplasm; GH, growth hormone therapy*.

By geographic regions based on the all-cause SMR, the association between pediatric somatropin treatment and the all-cause mortality was not statistically significant in Europe (SMR = 1.92, 95% CI: 0.71–5.23, *P* = 0.202; *I*^2^ = 99.4%; *Tau*^2^ = 1.938) and Asia (SMR = 1.90, 95% CI: 0.39–9.17, *P* = 0.424; *I*^2^ = 80.6%; *Tau*^2^ = 1.044) and also no significance was detected between rhGH therapy and cancer mortality in children in Europe (SMR = 1.47, 95% CI: 0.73–2.96, *P* = 0.279; *I*^2^ = 59.3%; *Tau*^2^ = 0.227) based on the geographical areas of cancer SMR. Based on cancer SIR by geographic regions, the association was nonsignificant between rhGH therapy and standard cancer incidence in both Europe (SIR = 1.09, 95% CI: 0.08–14.50, *P* = 0.951; *I*^2^ = 98.8%; *Tau*^2^ = 8.071) and Asia (SIR = 2.09, 95% CI: 0.37–11.81, *P* = 0.058; *I*^2^ = 65.8%; *Tau*^2^ = 1.046). The association between childhood rhGH therapy and second neoplasm was statistically significant in North America (RR = 2.20, 95% CI: 1.61–3.02, *P* < 0.001; *I*^2^ = 0.00%; *Tau*^2^ = 0.000). However, the statistical significance was not demonstrated in Europe (RR = 1.57, 95% CI: 0.93–2.66, *P* = 0.094; *I*^2^ = 12.9%; *Tau*^2^ = 0.051).

By gender based on the all-cause SMR, the association between rhGH therapy and all-cause mortality was not statistically significant in either boys (SMR = 2.50, 95% CI: 0.81–7.69, *P* = 0.110; *I*^2^ = 99.4%; *Tau*^2^ = 1.629) or girls (SMR = 3.01, 95% CI: 0.71–12.78, *P* = 0.135; *I*^2^ = 99.2%; *Tau*^2^ = 2.663).

By study design based on the all-cause SMR, the association between rhGH therapy and all-cause mortality in children was not statistically significant in prospective cohorts (SMR = 1.16, 95% CI: 0.44–3.10, *P* = 0.765; *I*^2^ = 99.4%; *Tau*^2^ = 1.629) and in retrospective cohorts, the SMR for rhGH therapy and all-cause mortality was 1.56 (95% CI: 1.02–2.38, *P* = 0.041; *I*^2^ = 60.6%; *Tau*^2^ = 0.102). Based on the study type of cancer SMR, there was no statistical significance between growth hormone therapy and tumor mortality in retrospective cohorts (SMR = 1.47, 95% CI: 0.73–2.96, *P* = 0.279; *I*^2^ = 59.3%; *Tau*^2^ = 0.227). Based on the study design of cancer SIR, in prospective cohorts, there was no statistical significance between rhGH therapy and the standard incidence of tumor (SIR =1.22, 95% CI: 0.15–10.31, *P* = 0.853; *I*^2^ = 99.1%; *Tau*^2^ = 5.286), yet significance was attained in retrospective cohorts (SIR = 1.53, 95% CI: 1.02–2.31, *P* = 0.040; *I*^2^ = 75.8%; *Tau*^2^ = 0.286). By the study design based on the second neoplasm, the association between rhGH therapy and second neoplasm reached statistical significance (RR = 1.77, 95% CI: 1.33–2.35, *P* < 0.001; *I*^2^ = 26.7%; *Tau*^2^ = 0.055).

By risk based on all-cause SMR, in children with low risk (SMR = 1.25, 95% CI: 1.17–1.34, *P* < 0.001; *I*^2^ = 14.4%; *Tau*^2^ = 0.009), moderate risk (SMR = 4.00, 95% CI: 3.50–4.57, *P* < 0.001; *I*^2^ = 69.0%; *Tau*^2^ = 0.061), or high risk (SMR = 16.88, 95% CI: 14.52–19.63, *P* < 0.001; *I*^2^ = 0.1%; *Tau*^2^ = 0.101), the relationship between rhGH therapy and all-cause mortality was statistically significant. Based on the risk of cancer SMR, there was statistical significance between rhGH therapy and cancer mortality in children with not-high risk (SMR = 8.28, 95% CI: 1.62–42.41, *P* = 0.011; *I*^2^ = 99.6%; *Tau*^2^ = 2.714). In addition, rhGH therapy did not significantly affect standard tumor incidence among children at not-high risk based on the risk of cancer SIR (SIR = 1.88, 95% CI: 0.99–3.57, *P* = 0.055; *I*^2^ = 96.9%; *Tau*^2^ = 0.602).

By duration of rhGH therapy based on the all-cause SMR, the association between rhGH therapy and all-cause mortality was not statistically significant when treatment duration was ≥5 years (SMR = 1.96, 95% CI: 0.83–4.65, *P* = 0.126; *I*^2^ = 95.8%; *Tau*^2^ = 1.427). However, when the treatment time was <5 years (SMR =3.20, 95% CI: 1.78–5.76, *P* < 0.001; *I*^2^ = 98.2%; *Tau*^2^ = 1.665), the association was significant.

By overall rhGH exposure dose based on all-cause SMR, the association between rhGH therapy and all-cause mortality was not statistically significant when rhGH exposure during childhood was <25 mg/kg (SMR = 2.03, 95% CI: 0.62–6.59, *P* = 0.241; *I*^2^ = 98.8%; *Tau*^2^ = 2.493), 25–50 mg/kg (SMR = 2.85, 95% CI: 0.89–9.09, *P* = 0.077; *I*^2^ = 98.3%; *Tau*^2^ = 1.711), and 50–100 mg/kg (SMR = 2.64, 95% CI: 0.81–8.55, *P* = 0.106; *I*^2^ = 96.9%; *Tau*^2^ = 1.341), whereas the association was statistically significant when total rhGH exposure was ≥100 mg/kg (SMR = 3.32, 95% CI: 1.22–9.08, *P* = 0.019; *I*^2^ = 85.8%; *Tau*^2^ = 0.832).

By follow-up period based on all-cause SMR, there was no statistically significant association between rhGH therapy and all-cause mortality when the follow-up period ≥10 years (SMR = 0.98, 95% CI: 0.75–1.29, *P* = 0.899; *I*^2^ = 78.3%; *Tau*^2^ = 0.127). The association between rhGH therapy and all-cause mortality was statistically significant in studies with follow-up duration <10 years (SMR = 4.62, 95% CI: 1.19–18.01, *P* = 0.027; *I*^2^ = 99.6%; *Tau*^2^ = 1.435). The association between rhGH therapy and cancer mortality was not statistically significant in studies with follow-up duration ≥10 years (SMR = 2.59, 95% CI: 0.55–12.09, *P* = 0.228; *I*^2^ = 96.7%; *Tau*^2^ = 2.361) based on cancer SMR. Based on cancer SIR of follow-up, there was no statistically significant association between rhGH therapy and standard cancer incidence at follow-up times ≥10 years (SIR =1.54, 95% CI: 0.68–3.47, *P* < 0.001; *I*^2^ = 97.5%; *Tau*^2^ = 2.287). Nevertheless, there was a statistically significant relationship between second neoplasm and rhGH therapy (RR = 1.77, 95% CI: 1.33–2.35, *P* < 0.001; *I*^2^ = 26.7%; *Tau*^2^ = 0.055).

## Discussion

To the best of our knowledge, this is, thus far the most comprehensive meta-analysis that has examined the association between rhGH therapy during childhood and multiple clinical outcomes including the all-cause mortality, cancer mortality, standard cancer incidence, and second neoplasm. Our key findings suggested that rhGH therapy in childhood had no deleterious effects on all-cause mortality, cancer mortality, and standard cancer incidence. In contrast, rhGH therapy was a risk factor for the development of the second neoplasm. Furthermore, our analyses suggested that differences in the geographic region, gender, treatment duration, mean rhGH dose, overall rhGH exposure dose, and initial disease accounted for heterogeneity. Our findings highlight the relative safety of growth hormone use in childhood and provide high-quality evidence for pediatrics, particularly for these conditions requiring rhGH therapy.

Extending the findings of previous individual studies that assessed only one or two clinical outcomes after rhGH therapy, we, in this meta-analysis, comprehensively evaluated all the possible outcomes in both the overall analyses and subgroup analyses. It is worth noting that all-cause mortality and cancer incidence were significantly higher than expected in the low- and intermediate-risk groups. Although only 2 articles were involved in the analysis of total exposure to rhGH in children, the all-cause mortality rate was significantly higher than expected when the total exposure dose was over 100 mg/kg based on the results of the analysis of the overall exposure dose. However, this dose needs to be determined by future studies. Moreover, we also interestingly noticed that all-cause mortality was significantly lower than expected for both boys and girls. Although the exact mechanisms behind these positive findings are not fully understood, we agree that further well-designed, long-term studies are warranted to further enrich our understanding of the clinical implication of rhGH therapy in childhood in future mortality risk in adulthood.

The current meta-analysis is based on the previous meta-analysis conducted by Deodati et al. (12) by pooling the results of 12 studies, who found no significant increase in the malignant tumor SMRs, yet overall cancer SIRs (2.74; 95% CI: 1.18–4.41) and RRs of second tumors (1.99; 95% CI: 1.28–3.08) were significantly increased. In the present meta-analysis, by contrast, we found that all-cause mortality and malignancy incidence were significantly lower than expected, that is, rhGH therapy was not a risk factor for all-cause mortality and malignancy incidence. The reasons for the conflicting observations between the meta-analysis by Deodati et al. (12) and this meta-analysis are mainly because of the power to detect statistical significance, as we incorporated the results from 24 articles.

Our finding that no significant association was found between the dose of rhGH and mortality and cancer incidence makes causality less likely. However, some studies have reported an increased incidence of bone cancer and bladder cancer in patients treated with rhGH and in patients with Hodgkin lymphoma (2, 25). Nonetheless in this meta-analysis, we did not conduct relevant subgroup analysis due to a lack of data on the initial disease of patients with detailed types of cancers. In addition, we believe that rhGH therapy should be carried out with caution in high-risk patients and that the start of rhGH therapy should be carefully discussed (34).

Available evidence suggests an increased risk of secondary tumors in rhGH recipients. Growth hormone is potent mitosis and anti-apoptotic hormone, and increased activity of the growth hormone/IGF-I axis is associated with an increased risk of cancer (35). Therefore, with the use of growth hormone therapy, the researchers' vigilance against the potential cancer risk accompanied this treatment from the beginning. Animal experiments showed that in spontaneous pygmy rats lacking rhGH, the administration of the carcinogen N-methyl-N-nitrosourea did not induce breast tumors. However, the tumors were developed in GH-treated rats (36). Moreover, after stopping hormone replacement, almost all the tumors have completely degenerated in animal models of rhGH receptor knockout mice hybridizing with Tag mice prone to prostate tumors, and similar findings were described by other investigators (35, 37). High IGF-1 or high growth hormone levels may induce messenger RNA alterations or other molecular changes and angiogenesis and inhibit apoptosis. This may further stimulate the carcinogenic potential that already exists (33, 38, 39). Molecular signaling pathways that affect cell proliferation, differentiation, and survival are regulated by the GH-IGF-1 axis. The carcinogenic process interacts with GH-IGF-1 signaling pathways, employs these physiological signaling pathways, and converts them into abnormal signaling pathways (33, 38).

Generally, the findings of this meta-analysis are reassuring, but some biases, confounding factors, and weaknesses limit the value and interpretation of all data reported to date. Detailed information on dosage, duration of treatment, and primary disease in children need more literature support and although the relevant subgroup analysis was conducted in this study, the number of relevant articles was relatively small. Future prospective studies are also needed to confirm these results and answer more difficult questions about the appropriate period to start GH therapy after achieving complete remission, and how to deal with children with “chronic” low-grade tumor diseases and growth hormone deficiency (GHD). In addition, more research is required on the optimal dosage of rhGH therapy (34).

## Conclusion

Our findings indicate that rhGH therapy is not related to all-cause mortality, cancer mortality, and cancer incidence, yet it seems to trigger a second tumor risk. The long-term safety of growth hormone therapy still deserves more attention as mortality from certain causes is increasing, and the need for long-term monitoring remains essential.

## Data Availability Statement

The original contributions presented in the study are included in the article/Supplementary Material, further inquiries can be directed to the corresponding author.

## Author Contributions

JH, ZZ, and WN: conceived and designed the experiments. MH, XW, and ZZ: performed the experiments. MH, XD, and JH: analyzed the data and contributed materials/analysis tools. MH, XW, JH, and WN: wrote the article. All the authors read and approved the final manuscript before submission.

## Conflict of Interest

The authors declare that the research was conducted in the absence of any commercial or financial relationships that could be construed as a potential conflict of interest.

## Publisher's Note

All claims expressed in this article are solely those of the authors and do not necessarily represent those of their affiliated organizations, or those of the publisher, the editors and the reviewers. Any product that may be evaluated in this article, or claim that may be made by its manufacturer, is not guaranteed or endorsed by the publisher.
