# Supplementary material for: Association Between Recombinant Growth Hormone Therapy and All-Cause Mortality and Cancer Risk in Childhood: Systematic Review and Meta-Analysis
Source: Front Pediatr. 2022 Apr 22;10:866295. doi: 10.3389/fped.2022.866295 (PMC9073080; doi:10.3389/fped.2022.866295)
Supplement: Supplementary file 2 [file Table_2.pdf]

Supplementary Table 2. Main characteristics of the studies conducted on r-hGH therapy and mortality, cancer risk included in the meta-analysis.

Year	First author	Gender	Age at start GH treatment(years)	Follow up (years)	GH dose	Duration of treatment (years)	Estimate	Risk	Initial Diagnosis	Treatment duration (years)	Mean GH dose	Overall exposure	Estimate size	Low	High
2002	Wing Leung	both	10.9 (6.9-14.7)	15.6 (7.3-22.1)	0.3 mg/kg/week	4.5 (1-8)	RR SN after leukemia	_	_	_	_	_	1.58	0.38	6.53
2002	Sklar	both	10 (3.1-20.8)	6.2 (0.4-20.6)	_	4.6 (0.1-14)	RR SN after leukemia	_	_	_	_	_	4.98	1.95	12.74
2002	Sklar	both	10 (3.1-20.9)	6.2 (0.4-20.6)	_	4.6 (0.1-14)	RR SN after CSN tumor	_	_	_	_	_	2.34	0.96	5.70
2002	Swerdlow	both	_	21.2	_	_	Cancer SMR	non-high	_	_	_	_	2.3	0.80	5.00
2002	Swerdlow	both	_	16.1	_	_	Cancer SIR	non-high	_	_	_	_	1.4	0.50	2.80
2002	Swerdlow	both	_	21.2	_	_	Cancer SMR	all	_	_	_	_	2.8	1.30	5.10
2002	Swerdlow	both	_	16.1	_	_	Cancer SIR	all	_	_	_	_	1.7	0.90	2.90
2006	Ergun-Longmire	both	11 (1-20.8)	8.86	_	4.6 (0.1-14)	RR SN	_	_	_	_	_	2.15	1.33	3.47
2006	Ergun-Longmire	both	12 (1-20.8)	8.86	_	4.6 (0.1-14)	RR SN after leukemia	_	_	_	_	_	2.3	0.90	5.80
2006	Ergun-Longmire	both	13 (1-20.8)	8.86	_	4.6 (0.1-14)	RR SN after CSN tumor	_	_	_	_	_	1.42	0.67	3.02
2010	Bell	both	_	21	_	3.6	Cancer SIR	non-high	_	_	_	_	1.12	0.75	1.61
2010	Wilton	both	_	21	0.26 (0.12-0.48) mg/kg/week	3.6 (0.08-9.70)	Cancer SIR	non-high	_	_	_	_	1.26	0.86	1.78
2011	Child	both	46.4 (34.1-56.3)	3.7 ± 2.9	0.30 (0.20–0.46) mg/day	_	Cancer SIR	_	_	_	_	_	2.74	1.18	5.41
2011	Mackenzie	both	33 (14-45)	14.5	_	8 (4-10)	RR SN	_	_	_	_	_	2.5	0.50	12.61
2012	Carel	both	11.0 ± 3.4	17.3 ± 4.1	24.6 ± 12.2 μg/kg/d	3.9±2.6	All cause SMR	low	_	_	_	_	1.33	1.08	1.64
2012	Carel	female	11.0 ± 3.4	17.3 ± 4.1	24.6 ± 12.2 μg/kg/d	3.9±2.6	All cause SMR	low	_	_	_	_	1.21	0.65	2.08
2012	Carel	male	11.0 ± 3.4	17.3 ± 4.1	24.6 ± 12.2 μg/kg/d	3.9±2.6	All cause SMR	low	_	_	_	_	1.36	1.08	1.69
2012	Carel	both	11.0 ± 3.4	17.3 ± 4.1	24.6 ± 12.2 μg/kg/d	3.9±2.6	All cause SMR	low	_	_	0-20 μg/kg/d	_	1.07	0.74	1.52
2012	Carel	both	11.0 ± 3.4	17.3 ± 4.1	24.6 ± 12.2 μg/kg/d	3.9±2.6	All cause SMR	low	_	_	20-30 μg/kg/d	_	1.2	0.84	1.67
2012	Carel	both	11.0 ± 3.4	17.3 ± 4.1	24.6 ± 12.2 μg/kg/d	3.9±2.6	All cause SMR	low	_	_	30-50 μg/kg/d	_	1.41	0.46	3.30
2012	Carel	both	11.0 ± 3.4	17.3 ± 4.1	24.6 ± 12.2 μg/kg/d	3.9±2.6	All cause SMR	low	_	_	>50 μg/kg/d	_	3.41	1.25	7.42

2012	Carel	both	11.0 ± 3.4	17.3 ± 4.1	24.6 ± 12.2 μg/kg/d	3.9±2.6	All cause SMR	low	_	0-2	_	_	1.83	1.22	2.62
2012	Carel	both	11.0 ± 3.4	17.3 ± 4.1	24.6 ± 12.2 μg/kg/d	3.9±2.6	All cause SMR	low	_	2-4	_	_	1.21	0.85	1.68
2012	Carel	both	11.0 ± 3.4	17.3 ± 4.1	24.6 ± 12.2 μg/kg/d	3.9±2.6	All cause SMR	low	_	>4	_	_	1.23	0.80	1.80
2012	Carel	both	11.0 ± 3.4	17.3 ± 4.1	24.6 ± 12.2 μg/kg/d	3.9±2.6	All cause SMR	low	_	_	_	<16 mg/kg	1.29	0.81	1.96
2012	Carel	both	11.0 ± 3.4	17.3 ± 4.1	24.6 ± 12.2 μg/kg/d	3.9±2.6	All cause SMR	low	_	_	_	16-27 mg/kg	0.95	0.55	1.51
2012	Carel	both	11.0 ± 3.4	17.3 ± 4.1	24.6 ± 12.2 μg/kg/d	3.9±2.6	All cause SMR	low	_	_	_	27-47 mg/kg	1.1	0.64	1.75
2012	Carel	both	11.0 ± 3.4	17.3 ± 4.1	24.6 ± 12.2 μg/kg/d	3.9±2.6	All cause SMR	low	_	_	_	>=47 mg/kg	1.84	1.13	2.84
2012	Carel	both	11.0 ± 3.4	17.3 ± 4.1	24.6 ± 12.2 μg/kg/d	3.9±2.6	Cancer SMR	low	_	_	_	_	1.02	0.41	2.09
2013	Woodmansee	both	10.8 (8.9-12.9)	<3	0.22 ± 0.03 mg/kg/week	2.9 (1.4-4.8)	RR SN	_	_	_	_	_	1.3	0.14	4.80
2014	Mo	both	11.3 ± 7.2	3.7 ± 3.3	0.54 ± 0.33 mg/kg/week	7.5±4.3	All cause SMR	_	_	_	_	_	1.14	0.55	2.10
2014	Mo	both	10 ± 6.1	4.2 ± 3.6	0.54 ± 0.30 mg/kg/week	8±5.1	All cause SMR	_	_	_	_	_	0.33	0.01	1.84
2014	Mo	both	11.3 ± 7.2	3.7 ± 3.3	0.54 ± 0.33 mg/kg/week	7.5±4.3	cancer SIR	_	_	_	_	_	0.27	0.01	1.50
2014	Mo	both	10 ± 6.1	4.2 ± 3.6	0.54 ± 0.30 mg/kg/week	8±5.1	cancer SIR	_	_	_	_	_	0	0.00	2.45
2014	Patterson	both	<15	_	_	_	RR SN	_	_	_	_	_	1	0.60	1.80
2015	Brignardello	both	<18	11	0.14-0.28 mg/kg/week	>1	RR SN	_	_	_	_	_	3.74	0.85	16.43
2016	Albertsson-Wikland	both	<18	0-37.5	36 μg/kg/d	_	All cause SMR	_	_	_	_	_	1.43	0.89	2.19
2016	Child	both	9.5 ± 4.0	3.4	0.26 (0.20 – 0.32) mg/kg/week	3.4 ± 2.5	Cancer SIR	_	_	_	_	_	1.02	0.54	1.75
2016	Libruder	both	8.8 ± 3.8	9.5 ± 4.0	_	_	All cause SMR	low	_	_	_	_	0.81	0.22	2.08
2016	Libruder	both	8.8 ± 3.8	6.5 ± 4.0	_	_	Cancer SIR	low	_	_	_	_	0.76	0.09	2.73
2016	Libruder	both	7.7 ± 4.4	11.1 ± 4.6	_	_	All cause SMR	moderate	_	_	_	_	4.05	1.62	3.84
2016	Libruder	both	7.7 ± 4.4	8.1 ± 4.6	_	_	Cancer SIR	moderate	_	_	_	_	4.52	1.22	11.57
2017	Quigley	both	8.5 ± 3.7	>=4	0.26 ± 0.10 mg/kg/week	7.3±3.3	All cause SMR	_	_	_	_	_	0.77	0.56	1.05
2017	Quigley	both	8.5 ± 3.7	>=4	0.26 ± 0.10 mg/kg/week	7.3±3.3	All cause SMR	_	_	_	_	_	0.11	0.02	0.33
2017	Swerdlow	both	<19	16.5	_	_	Cancer SMR	non-high	_	_	_	_	13.7	12.10	15.50
2017	Swerdlow	both	<19	14.8	_	_	Cancer SIR	non-high	_	_	_	_	2.2	1.90	2.60
2017	Swerdlow	both	<19	16.5	_	_	Cancer SMR	non-high	cancer	_	_	_	101.9	89.60	116.00

2017	Swerdlow	both	<19	14.8	_	_	Cancer SIR	non-high	cancer	_	_	_	7.6	6.10	9.60
2017	Swerdlow	both	<19	16.5	_	_	Cancer SMR	non-high	non-cancer	_	_	_	1.3	0.90	2.00
2017	Swerdlow	both	<19	14.8	_	_	Cancer SIR	non-high	non-cancer	_	_	_	1.2	1.00	1.60
2018	Krzyzanowska-Mittermayer	both	10.4 ± 4.4	5.9	_	_	Cancer SIR	_	_	_	_	_	6.52	2.97	12.37
2018	Krzyzanowska-Mittermayer	both	10.4 ± 4.4	5.9	_	_	Cancer SIR	_	_	_	_	_	0.47	0.09	1.37
2018	Poidvin	both	11.0 ± 3.4	17.4 ± 5.3	24.5 ± 12.3 µg/kg/d	3.9 ± 2.6	Cancer SIR	_	_	_	_	_	0.8	0.50	1.20
2018	Poidvin	both	9.7 ± 3.8	17.4 ± 5.3	24.5 ± 12.3 µg/kg/d	3.9 ± 2.6	Cancer SMR	_	_	_	_	_	1	0.40	2.10
2019	Child	both	11.0 ± 3.5	4.3 ± 3.1	0.27 ± 0.10 mg/kg/week	_	All cause SMR	_	_	_	_	_	0.61	0.44	0.82
2019	Child	both	11.0 ± 3.5	7.1 ± 2.6	0.27 ± 0.10 mg/kg/week	_	All cause SMR	_	_	_	_	_	0.81	0.58	1.10
2019	Child	both	11.0 ± 3.5	_	0.27 ± 0.10 mg/kg/week	_	Cancer SIR	_	_	_	_	_	0.71	0.39	1.20
2019	Swerdlow	both	<19	14.9	_	_	Cancer SIR	_	_	_	_	_	75.4	54.90	103.60
2019	Swerdlow	both	<19	14.9	_	_	Cancer SIR	_	_	_	_	_	466.3	337.80	643.50
2019	Swerdlow	both	<19	14.9	_	_	Cancer SIR	_	non-cancer	_	_	_	2.4	0.30	16.70
2020	Sävendahl	both	10.9 ± 3.3	16.3 ± 4.8	26.3 ± 11 µg/kg/d	4.5 ± 3.0	All cause SMR	low risk	_	_	_	_	1.1	0.90	1.30
2020	Sävendahl	both	10.0 ± 3.5	17.2 ± 4.6	33.3 ± 17.4 µg/kg/d	4.8 ± 3.1	All cause SMR	low risk	_	_	_	_	1.5	1.10	1.90
2020	Sävendahl	both	9.9 ± 3.9	17 ± 5	35 ± 10.8 µg/kg/d	6 ± 3.6	All cause SMR	moderate risk	_	_	_	_	3.8	3.30	4.40
2020	Sävendahl	both	11.1 ± 3.2	15.4 ± 5.6	25.6 ± 8.6 µg/kg/d	4.8 ± 3.1	All cause SMR	high risk	_	_	_	_	1.71	15.60	18.70
2020	Sävendahl	male	10.9 ± 3.3	16.3 ± 4.8	26.3 ± 11 µg/kg/d	4.5 ± 3.0	All cause SMR	low risk	_	_	_	_	1.1	0.90	1.30
2020	Sävendahl	male	10.0 ± 3.5	17.2 ± 4.6	33.3 ± 17.4 µg/kg/d	4.8 ± 3.1	All cause SMR	low risk	_	_	_	_	1.5	1.10	2.00
2020	Sävendahl	male	9.9 ± 3.9	17 ± 5	35 ± 10.8 µg/kg/d	6 ± 3.6	All cause SMR	moderate risk	_	_	_	_	3.4	2.70	4.20
2020	Sävendahl	male	11.1 ± 3.2	15.4 ± 5.6	25.6 ± 8.6 µg/kg/d	4.8 ± 3.1	All cause SMR	high risk	_	_	_	_	12.7	11.20	14.30
2020	Sävendahl	female	10.9 ± 3.3	16.3 ± 4.8	26.3 ± 11 µg/kg/d	4.5 ± 3.0	All cause SMR	low risk	_	_	_	_	1	0.60	1.80
2020	Sävendahl	female	10.0 ± 3.5	17.2 ± 4.6	33.3 ± 17.4 µg/kg/d	4.8 ± 3.1	All cause SMR	low risk	_	_	_	_	1.3	0.70	2.50
2020	Sävendahl	female	9.9 ± 3.9	17 ± 5	35 ± 10.8 µg/kg/d	6 ± 3.6	All cause SMR	moderate risk	_	_	_	_	4.3	3.60	5.30

2020	Sävendahl	female	11.1 ± 3.2	15.4 ± 5.6	25.6 ± 8.6 μg/kg/d	4.8 ± 3.1	All cause SMR	high risk	_	_	_	_	33.2	28.80	38.30
2020	Sävendahl	both	10.9 ± 3.3	16.3 ± 4.8	26.3 ± 11 μg/kg/d	4.5 ± 3.0	All cause SMR	low risk	_	<2	_	_	1.6	1.10	2.30
2020	Sävendahl	both	10.0 ± 3.5	17.2 ± 4.6	33.3 ± 17.4 μg/kg/d	4.8 ± 3.1	All cause SMR	low risk	_	<2	_	_	2.5	1.50	4.20
2020	Sävendahl	both	9.9 ± 3.9	17 ± 5	35 ± 10.8 μg/kg/d	6 ± 3.6	All cause SMR	moderate risk	_	<2	_	_	9.5	7.20	12.60
2020	Sävendahl	both	11.1 ± 3.2	15.4 ± 5.6	25.6 ± 8.6 μg/kg/d	4.8 ± 3.1	All cause SMR	high risk	_	<2	_	_	32.6	27.40	38.80
2020	Sävendahl	both	10.9 ± 3.3	16.3 ± 4.8	26.3 ± 11 μg/kg/d	4.5 ± 3.0	All cause SMR	low risk	_	2	_	_	0.8	0.50	1.40
2020	Sävendahl	both	10.0 ± 3.5	17.2 ± 4.6	33.3 ± 17.4 μg/kg/d	4.8 ± 3.1	All cause SMR	low risk	_	2	_	_	1.6	0.80	3.10
2020	Sävendahl	both	9.9 ± 3.9	17 ± 5	35 ± 10.8 μg/kg/d	6 ± 3.6	All cause SMR	moderate risk	_	2	_	_	4.9	3.20	7.60
2020	Sävendahl	both	11.1 ± 3.2	15.4 ± 5.6	25.6 ± 8.6 μg/kg/d	4.8 ± 3.1	All cause SMR	high risk	_	2	_	_	19.9	15.70	25.00
2020	Sävendahl	both	10.9 ± 3.3	16.3 ± 4.8	26.3 ± 11 μg/kg/d	4.5 ± 3.0	All cause SMR	low risk	_	3	_	_	1.1	0.70	1.80
2020	Sävendahl	both	10.0 ± 3.5	17.2 ± 4.6	33.3 ± 17.4 μg/kg/d	4.8 ± 3.1	All cause SMR	low risk	_	3	_	_	1.6	0.80	3.10
2020	Sävendahl	both	9.9 ± 3.9	17 ± 5	35 ± 10.8 μg/kg/d	6 ± 3.6	All cause SMR	moderate risk	_	3	_	_	4.7	3.20	7.00
2020	Sävendahl	both	11.1 ± 3.2	15.4 ± 5.6	25.6 ± 8.6 μg/kg/d	4.8 ± 3.1	All cause SMR	high risk	_	3	_	_	14.4	11.10	18.70
2020	Sävendahl	both	10.9 ± 3.3	16.3 ± 4.8	26.3 ± 11 μg/kg/d	4.5 ± 3.0	All cause SMR	low risk	_	4-5	_	_	0.9	0.50	1.50
2020	Sävendahl	both	10.0 ± 3.5	17.2 ± 4.6	33.3 ± 17.4 μg/kg/d	4.8 ± 3.1	All cause SMR	low risk	_	4-5	_	_	1.3	0.60	2.50
2020	Sävendahl	both	9.9 ± 3.9	17 ± 5	35 ± 10.8 μg/kg/d	6 ± 3.6	All cause SMR	moderate risk	_	4-5	_	_	3.5	2.50	5.00
2020	Sävendahl	both	11.1 ± 3.2	15.4 ± 5.6	25.6 ± 8.6 μg/kg/d	4.8 ± 3.1	All cause SMR	high risk	_	4-5	_	_	14.2	11.40	17.70
2020	Sävendahl	both	10.9 ± 3.3	16.3 ± 4.8	26.3 ± 11 μg/kg/d	4.5 ± 3.0	All cause SMR	low risk	_	6-9	_	_	1.1	0.60	1.90
2020	Sävendahl	both	10.0 ± 3.5	17.2 ± 4.6	33.3 ± 17.4 μg/kg/d	4.8 ± 3.1	All cause SMR	low risk	_	6-9	_	_	0.9	0.40	2.10
2020	Sävendahl	both	9.9 ± 3.9	17 ± 5	35 ± 10.8 μg/kg/d	6 ± 3.6	All cause SMR	moderate risk	_	6-9	_	_	3.8	2.80	5.10
2020	Sävendahl	both	11.1 ± 3.2	15.4 ± 5.6	25.6 ± 8.6 μg/kg/d	4.8 ± 3.1	All cause SMR	high risk	_	6-9	_	_	13.3	10.50	16.70
2020	Sävendahl	both	10.9 ± 3.3	16.3 ± 4.8	26.3 ± 11 μg/kg/d	4.5 ± 3.0	All cause SMR	low risk	_	>=10	_	_	0.7	0.30	1.90
2020	Sävendahl	both	10.0 ± 3.5	17.2 ± 4.6	33.3 ± 17.4 μg/kg/d	4.8 ± 3.1	All cause SMR	low risk	_	>=10	_	_	0.9	0.30	2.80
2020	Sävendahl	both	9.9 ± 3.9	17 ± 5	35 ± 10.8 μg/kg/d	6 ± 3.6	All cause SMR	moderate risk	_	>=10	_	_	0.8	0.40	1.60
2020	Sävendahl	both	11.1 ± 3.2	15.4 ± 5.6	25.6 ± 8.6 μg/kg/d	4.8 ± 3.1	All cause SMR	high risk	_	>=10	_	_	5.9	3.40	10.50
2020	Sävendahl	both	10.9 ± 3.3	16.3 ± 4.8	26.3 ± 11 μg/kg/d	4.5 ± 3.0	All cause SMR	low risk	_	_	<15 μg/kg/d	_	0.7	0.20	2.10

2020	Sävendahl	both	10.0 ± 3.5	17.2 ± 4.6	33.3 ± 17.4 µg/kg/d	4.8 ± 3.1	All cause SMR	low risk	_	_	<15 µg/kg/d	_	1.7	0.50	5.20
2020	Sävendahl	both	9.9 ± 3.9	17 ± 5	35 ± 10.8 µg/kg/d	6 ± 3.6	All cause SMR	moderate risk	_	_	<15 µg/kg/d	_	3.5	1.30	9.20
2020	Sävendahl	both	11.1 ± 3.2	15.4 ± 5.6	25.6 ± 8.6 µg/kg/d	4.8 ± 3.1	All cause SMR	high risk	_	_	<15 µg/kg/d	_	20.5	15.00	28.00
2020	Sävendahl	both	10.9 ± 3.3	16.3 ± 4.8	26.3 ± 11 µg/kg/d	4.5 ± 3.0	All cause SMR	low risk	_	_	15-19 µg/kg/d	_	1	0.70	1.60
2020	Sävendahl	both	10.0 ± 3.5	17.2 ± 4.6	33.3 ± 17.4 µg/kg/d	4.8 ± 3.1	All cause SMR	low risk	_	_	15-19 µg/kg/d	_	1.2	0.60	2.50
2020	Sävendahl	both	9.9 ± 3.9	17 ± 5	35 ± 10.8 µg/kg/d	6 ± 3.6	All cause SMR	moderate risk	_	_	15-19 µg/kg/d	_	4.5	2.70	7.40
2020	Sävendahl	both	11.1 ± 3.2	15.4 ± 5.6	25.6 ± 8.6 µg/kg/d	4.8 ± 3.1	All cause SMR	high risk	_	_	15-19 µg/kg/d	_	15.9	12.30	20.60
2020	Sävendahl	both	10.9 ± 3.3	16.3 ± 4.8	26.3 ± 11 µg/kg/d	4.5 ± 3.0	All cause SMR	low risk	_	_	20-24 µg/kg/d	_	0.8	0.40	1.40
2020	Sävendahl	both	10.0 ± 3.5	17.2 ± 4.6	33.3 ± 17.4 µg/kg/d	4.8 ± 3.1	All cause SMR	low risk	_	_	20-24 µg/kg/d	_	1.8	0.90	3.40
2020	Sävendahl	both	9.9 ± 3.9	17 ± 5	35 ± 10.8 µg/kg/d	6 ± 3.6	All cause SMR	moderate risk	_	_	20-24 µg/kg/d	_	5	3.40	7.50
2020	Sävendahl	both	11.1 ± 3.2	15.4 ± 5.6	25.6 ± 8.6 µg/kg/d	4.8 ± 3.1	All cause SMR	high risk	_	_	20-24 µg/kg/d	_	13.7	10.90	17.10
2020	Sävendahl	both	10.9 ± 3.3	16.3 ± 4.8	26.3 ± 11 µg/kg/d	4.5 ± 3.0	All cause SMR	low risk	_	_	25-29 µg/kg/d	_	1	0.50	1.90
2020	Sävendahl	both	10.0 ± 3.5	17.2 ± 4.6	33.3 ± 17.4 µg/kg/d	4.8 ± 3.1	All cause SMR	low risk	_	_	25-29 µg/kg/d	_	1.2	0.50	2.80
2020	Sävendahl	both	9.9 ± 3.9	17 ± 5	35 ± 10.8 µg/kg/d	6 ± 3.6	All cause SMR	moderate risk	_	_	25-29 µg/kg/d	_	3.6	2.30	5.50
2020	Sävendahl	both	11.1 ± 3.2	15.4 ± 5.6	25.6 ± 8.6 µg/kg/d	4.8 ± 3.1	All cause SMR	high risk	_	_	25-29 µg/kg/d	_	16.8	13.30	21.30
2020	Sävendahl	both	10.9 ± 3.3	16.3 ± 4.8	26.3 ± 11 µg/kg/d	4.5 ± 3.0	All cause SMR	low risk	_	_	30-34 µg/kg/d	_	1	0.50	2.10
2020	Sävendahl	both	10.0 ± 3.5	17.2 ± 4.6	33.3 ± 17.4 µg/kg/d	4.8 ± 3.1	All cause SMR	low risk	_	_	30-34 µg/kg/d	_	1.7	0.80	3.30
2020	Sävendahl	both	9.9 ± 3.9	17 ± 5	35 ± 10.8 µg/kg/d	6 ± 3.6	All cause SMR	moderate risk	_	_	30-34 µg/kg/d	_	3.4	2.20	5.20
2020	Sävendahl	both	11.1 ± 3.2	15.4 ± 5.6	25.6 ± 8.6 µg/kg/d	4.8 ± 3.1	All cause SMR	high risk	_	_	30-34 µg/kg/d	_	22.2	17.00	29.00
2020	Sävendahl	both	10.9 ± 3.3	16.3 ± 4.8	26.3 ± 11 µg/kg/d	4.5 ± 3.0	All cause SMR	low risk	_	_	35-39 µg/kg/d	_	0.9	0.20	3.50
2020	Sävendahl	both	9.9 ± 3.9	17 ± 5	35 ± 10.8 µg/kg/d	6 ± 3.6	All cause SMR	moderate risk	_	_	35-39 µg/kg/d	_	3	1.80	4.80
2020	Sävendahl	both	11.1 ± 3.2	15.4 ± 5.6	25.6 ± 8.6 µg/kg/d	4.8 ± 3.1	All cause SMR	high risk	_	_	35-39 µg/kg/d	_	20	12.70	31.30
2020	Sävendahl	both	10.9 ± 3.3	16.3 ± 4.8	26.3 ± 11 µg/kg/d	4.5 ± 3.0	All cause SMR	low risk	_	_	40-49 µg/kg/d	_	1.7	0.50	5.20
2020	Sävendahl	both	9.9 ± 3.9	17 ± 5	35 ± 10.8 µg/kg/d	6 ± 3.6	All cause SMR	moderate risk	_	_	40-49 µg/kg/d	_	4.2	2.90	6.10
2020	Sävendahl	both	11.1 ± 3.2	15.4 ± 5.6	25.6 ± 8.6 µg/kg/d	4.8 ± 3.1	All cause SMR	high risk	_	_	40-49 µg/kg/d	_	9.9	5.20	19.10
2020	Sävendahl	both	10.0 ± 3.5	17.2 ± 4.6	33.3 ± 17.4 µg/kg/d	4.8 ± 3.1	All cause SMR	low risk	_	_	>=50 µg/kg/d	_	2.7	1.40	5.40

2020	Sävendahl	both	9.9 ± 3.9	17 ± 5	35 ± 10.8 µg/kg/d	6 ± 3.6	All cause SMR	moderate risk	_	_	>=50 µg/kg/d	_	5.1	2.70	9.50
2020	Sävendahl	both	11.1 ± 3.2	15.4 ± 5.6	25.6 ± 8.6 µg/kg/d	4.8 ± 3.1	All cause SMR	high risk	_	_	>=50 µg/kg/d	_	27.2	13.60	54.30
2020	Sävendahl	both	10.9 ± 3.3	16.3 ± 4.8	26.3 ± 11 µg/kg/d	4.5 ± 3.0	All cause SMR	low risk	_	_	_	<25 mg/kg	0.9	0.60	1.30
2020	Sävendahl	both	10.0 ± 3.5	17.2 ± 4.6	33.3 ± 17.4 µg/kg/d	4.8 ± 3.1	All cause SMR	low risk	_	_	_	<25 mg/kg	1.5	0.90	2.30
2020	Sävendahl	both	9.9 ± 3.9	17 ± 5	35 ± 10.8 µg/kg/d	6 ± 3.6	All cause SMR	moderate risk	_	_	_	<25 mg/kg	3.7	2.80	4.90
2020	Sävendahl	both	11.1 ± 3.2	15.4 ± 5.6	25.6 ± 8.6 µg/kg/d	4.8 ± 3.1	All cause SMR	high risk	_	_	_	<25 mg/kg	19.7	17.00	22.90
2020	Sävendahl	both	10.9 ± 3.3	16.3 ± 4.8	26.3 ± 11 µg/kg/d	4.5 ± 3.0	All cause SMR	low risk	_	_	_	25-49 mg/kg	0.9	0.60	1.50
2020	Sävendahl	both	10.0 ± 3.5	17.2 ± 4.6	33.3 ± 17.4 µg/kg/d	4.8 ± 3.1	All cause SMR	low risk	_	_	_	25-49 mg/kg	1.9	1.20	3.20
2020	Sävendahl	both	9.9 ± 3.9	17 ± 5	35 ± 10.8 µg/kg/d	6 ± 3.6	All cause SMR	moderate risk	_	_	_	25-49 mg/kg	3.6	2.60	4.80
2020	Sävendahl	both	11.1 ± 3.2	15.4 ± 5.6	25.6 ± 8.6 µg/kg/d	4.8 ± 3.1	All cause SMR	high risk	_	_	_	25-49 mg/kg	15.7	13.10	18.70
2020	Sävendahl	both	10.9 ± 3.3	16.3 ± 4.8	26.3 ± 11 µg/kg/d	4.5 ± 3.0	All cause SMR	low risk	_	_	_	50-99 mg/kg	1	0.60	1.90
2020	Sävendahl	both	10.0 ± 3.5	17.2 ± 4.6	33.3 ± 17.4 µg/kg/d	4.8 ± 3.1	All cause SMR	low risk	_	_	_	50-99 mg/kg	0.7	0.20	1.80
2020	Sävendahl	both	9.9 ± 3.9	17 ± 5	35 ± 10.8 µg/kg/d	6 ± 3.6	All cause SMR	moderate risk	_	_	_	50-99 mg/kg	3.9	2.90	5.30
2020	Sävendahl	both	11.1 ± 3.2	15.4 ± 5.6	25.6 ± 8.6 µg/kg/d	4.8 ± 3.1	All cause SMR	high risk	_	_	_	50-99 mg/kg	13.2	10.30	17.10
2020	Sävendahl	both	10.9 ± 3.3	16.3 ± 4.8	26.3 ± 11 µg/kg/d	4.5 ± 3.0	All cause SMR	low risk	_	_	_	>=100 mg/kg	0.8	0.20	3.30
2020	Sävendahl	both	10.0 ± 3.5	17.2 ± 4.6	33.3 ± 17.4 µg/kg/d	4.8 ± 3.1	All cause SMR	low risk	_	_	_	>=100 mg/kg	1.4	0.40	4.30
2020	Sävendahl	both	9.9 ± 3.9	17 ± 5	35 ± 10.8 µg/kg/d	6 ± 3.6	All cause SMR	moderate risk	_	_	_	>=100 mg/kg	4.4	2.90	6.50
2020	Sävendahl	both	11.1 ± 3.2	15.4 ± 5.6	25.6 ± 8.6 µg/kg/d	4.8 ± 3.1	All cause SMR	high risk	_	_	_	>=100 mg/kg	12.8	7.10	23.10
2020	Thomas-Teinturier	both	4 (0-13)	26 (5-41)	_	4 (0.2-14.5)	RR SN	_	_	_	_	_	1.3	0.90	2.00

Abbreviations: **r-hGH, Recombinant growth hormone therapy, SMR, standardized mortality ratios; SIR, standardized incidence ratio; RR, relative risk; SN, second neoplasm.**

(Continued)

Year	First author	Country	Sample size of patient	Study design	Adjustment
2002	Wing Leung	US	43	respective cohort	_
2002	Sklar	US or Canada	361	respective cohort	age at diagnosis, sex, radiation, and alkylating agent effects

2002	Swerdlow	UK	1848	respective cohort	age, sex, and calendar period
2006	Ergun-Longmire	US or Canada	361	respective cohort	age, sex, chemotherapy, alkylating agent score, and radiation
2010	Bell	US	54996	respective cohort	age
2010	Wilton	International	58603	respective cohort	age, sex, and country
2011	Child	International	6840	respective cohort	_
2011	Mackenzie	UK	110	respective cohort	_
2012	Carel	France	6560	respective cohort	year, age and sex
2013	Woodmansee	International	224	respective cohort	_
2014	Mo	France	1204	prospective cohort	_
2014	Patterson	International	338	retrospective cohort	sex; age at primary diagnosis; attained age at follow-up; cranial radiation dose/time since cranial radiation; and treatment with intrathecal methotrexate, estrogen, and/or progesterone treatment or alkylating agents
2015	Brignardello	Italy	26	retrospective cohort	age, sex and paediatric cancer type
2016	Albertsson-Wikland	Swedish	3847	retrospective cohort	age, sex and calendar year
2016	Child	International	19054	retrospective cohort	country, gender, race, age, and calendar year
2016	Libruder	Israel	2508	retrospective cohort	_
2017	Quigley	International	9504	prospective cohort	age, sex
2017	Swerdlow	International	23984	prospective cohort	_
2018	Krzyzanowska-Mittermayer	International	349	retrospective cohort	_
2018	Poidvin	France	6874	retrospective cohort	_
2019	Child	International	22311	prospective cohort	_
2019	Swerdlow	Europe	10403	prospective cohort	_
2020	Sävendahl	Europe	24232	prospective cohort	_
2020	Thomas-Teinturier	France	196	respective cohort	year of diagnosis of first cancer by class and radiation dose volume
