# Supplementary material for: Association Between Recombinant Growth Hormone Therapy and All-Cause Mortality and Cancer Risk in Childhood: Systematic Review and Meta-Analysis
Source: Front Pediatr. 2022 Apr 22;10:866295. doi: 10.3389/fped.2022.866295 (PMC9073080; doi:10.3389/fped.2022.866295)
Supplement: Supplementary file 3 [file Table_3.pdf]

Supplementary Table 3. The Newcastle-Ottawa Scale (NOS) for assessing the quality of all cohort studies involved in this meta-analysis.

First author	Published year	Representative of the exposed cohort	Selection of the non-exposed cohort	Ascertainment of exposed	Demonstration that outcome of interest was no present at start of study	Control for important cohort	Additional factors	Assessment of outcome	Follow up	Adequacy of follow up	Score
Wing Leung	2002	1	0	1	1	1	0	1	1	1	7
Sklar	2002	1	0	1	1	1	1	1	1	1	8
Swerdlow	2002	1	0	1	1	1	1	1	1	1	8
Ergun-Longmire	2006	1	0	1	1	1	1	1	1	1	8
Bell	2010	1	0	1	1	1	1	1	1	1	8
Wilton	2010	1	0	1	1	1	1	1	1	1	8
Child	2011	1	0	1	1	1	0	1	1	1	7
Mackenzie	2011	1	0	1	1	1	0	1	1	1	7
Carel	2012	1	0	1	1	1	1	1	1	1	8
Woodmansee	2013	1	0	1	1	1	0	1	1	1	7
Mo	2014	1	0	1	1	1	0	1	1	1	7
Patterson	2014	1	0	1	1	1	1	1	1	1	8
Brignardello	2015	1	0	1	1	1	0	1	1	1	7
Albertsson-Wikland	2016	1	0	1	1	1	1	1	1	1	8
Child	2016	1	0	1	1	1	1	1	1	1	8
Libruder	2016	1	0	1	1	1	0	1	1	1	7
Quigley	2017	1	0	1	1	1	1	1	1	1	8
Swerdlow	2017	1	0	1	1	1	0	1	1	1	7
Krzyzanowska-Mittermayer	2018	1	0	1	1	1	0	1	1	1	7
Poidvin	2018	1	0	1	1	1	0	1	1	1	7
Child	2019	1	0	1	1	1	0	1	1	1	7
Swerdlow	2019	1	0	1	1	1	0	1	1	1	7
Sävendahl	2020	1	0	1	1	1	0	1	1	1	7
Thomas-Teinturier	2020	1	0	1	1	1	1	1	1	1	8
